# Preparation of Ni-P Composite Coatings and Study on the Corrosion Resistance and Antifouling Properties in Low-Temperature Flue Gas Environment

**DOI:** 10.3390/ma18173939

**Published:** 2025-08-22

**Authors:** Changqi Lv, Shengxian Cao, Bo Zhao, Xingdong Yu

**Affiliations:** School of Automation Engineering, Northeast Electric Power University, Jilin 132012, China; lv_changqi@163.com (C.L.); zhaobo@neepu.edu.cn (B.Z.); striveyxd@163.com (X.Y.)

**Keywords:** Ni-Cu-P-SiO_2_ coating, chemical plating, low-temperature flue gas, antifouling, corrosion resistance

## Abstract

In industrial production, flue gas heat exchangers are often affected by the low-temperature condensation of industrial flue gas due to the influence of the working environment, resulting in serious ash deposition and corrosion. In order to solve this problem, in this study, we developed an ash deposition and corrosion monitoring system to compare the ash deposition prevention performance and corrosion resistance of different materials, as well as its influence on the heat transfer performance of different materials in the same environment. The following coatings were selected for the experiment (values in parentheses are the concentrations of the added compounds): ND, Q235, 316L, Ni-Cu (0.4 g/L)-P, Ni-P-SiO_2_ (40 g/L), Ni-Cu (0.4 g/L)-P-SiO_2_ (20 g/L), Ni-Cu (0.4 g/L)-P-SiO_2_ (40 g/L), and Ni-Cu (0.4 g/L)-P-SiO_2_ (60 g/L). The results show that the Ni-Cu (0.4 g/L)-P-SiO_2_ (40 g/L) coating has excellent corrosion resistance, while the Ni-Cu (0.4 g/L)-P-SiO_2_ (60 g/L) coating shows excellent antifouling performance. Through the comparative analysis of polarization curves, impedance spectra, and coupled corrosion experiments, the test materials were ranked as follows based on their corrosion resistance: 316L > Ni-Cu-P-SiO_2_ (40 g/L) > Ni-Cu-P-SiO_2_ (20 g/L) > Ni-P-SiO_2_ > Ni-Cu-P-SiO_2_ (60 g/L) > Ni-Cu-P > ND > Q235. It was also demonstrated that the new coated pipes were able to reduce the exhaust temperature below the dew point and maximize the recovery of energy from the exhaust gas. The acid–ash coupling mechanism of the coating in the flue gas environment was further analyzed, and an acid–ash coupling model based on Cu and SiO_2_ is proposed. This model analyzes the effect of the coating under the acid–ash coupling mechanism. Using coated tubes in heat exchangers helps to recover waste heat from coal-fired boilers, enhance heat exchange efficiency, extend the service life of heat exchangers, and reduce costs.

## 1. Introduction

According to statistics, in the past ten years fossil energy has continued to be dominant [1]. However, with the development of society, the transformation and optimization of the energy structure have become urgent [2,3]. We not only need to meet the growing demand for energy but must also consider environmental protection and sustainable development. In thermal power generation, the required coal-fired boiler flue gas temperature is 110–130 °C, but in reality, the actual flue gas temperature is often 10–30 °C higher than this range [4], and these high-temperature gases are rich in waste heat resources. As an efficient energy utilization device, the flue gas heat exchanger plays an important role in the optimization of the energy structure. By making full use of the waste heat in the flue gas, these exchangers can convert the heat energy in the exhaust gas into useful energy, realize energy recycling, and improve the efficiency of energy utilization. However, because the heat transfer medium on the gas side of the heat exchanger is flue gas containing fly ash, sticky substances, and corrosion products, issues such as fouling and low-temperature acid dew point corrosion are often encountered [5,6,7]. This will cause problems with the heat exchanger, such as a reduced heat exchange area, service life, and heat exchange efficiency, and may even cause serious safety accidents. The problem of dew point corrosion at low temperatures also restricts the continued reduction in exhaust gas temperature and prevents the effective recovery of waste heat from flue gas. Therefore, developing new heat exchanger materials that can effectively resist fouling and dew point corrosion at low temperatures would be highly beneficial.

In order to understand the process of acid condensation, Zuo et al. [8] expounded the concept of the low-temperature acid dew point and its production conditions in detail, while Wei [9] established a one-dimensional model for acid condensation according to the diffusion effect. In 2013, by developing a numerical calculation model of acid vapor, Han et al. [10] discovered that increasing the flue gas temperature can enhance the mass fraction of the acid solution and decrease the deposition rate. In 2017, Chen [11] and others found through experiments that with the decrease in temperature, the phenomenon of acid condensation gradually began to appear. Sulfuric acid condensation occurred at 80 °C, and at 40 °C, a large amount of HCl dissolves in the condensed water. The low-temperature corrosion phenomenon occurs at the same time as the problems of ash deposition and scaling. Chen et al. [12] expounded the conditions and growth mechanism of fly ash production and provided new ideas for preventing ash deposition. In 2016, Shi et al. [13] found that fly ash would agglomerate at a lower furnace wall temperature and revealed its agglomeration mechanism through further experiments. In 2017 [14], the effect of the organic combination of condensation, ash deposition, and heat transfer on heat transfer performance was explored at low temperatures. It was found that ash deposition would lead to a decrease in heat transfer area, a decrease in heat transfer performance, and an increase in channel flow resistance. In addition, the influence of acid and ash on heat transfer performance can be reduced by changing the shape and arrangement of heat exchangers [6,15,16].

The above analysis of ash deposition and corrosion mechanisms provides a scientific basis and new ideas for preventing ash deposition and controlling heat exchangers. In recent years, electroless plating has attracted much attention due to its excellent corrosion resistance, thermal stability, and low production cost. In particular, Ni-P coatings [17] are the most widely used, and their long application time, mature preparation process, and good thermal stability have solved many problems encountered in industrial applications. However, with the development of the modern chemical industry and the existence of pores in Ni-P coatings themselves [18], they cannot be adapted to all scenarios. On the basis of Ni-P coatings, the surface structure and chemical properties of the coating can be changed by using the characteristics of third-phase nanoparticles or metals, meaning that coatings can be adapted to different application scenarios. For example, the addition of Cu [19,20] can increase a coating’s hydrophobicity and corrosion resistance, a Ni-P-Al_2_O_3_ coating [21] prepared on the surface of an alloy can increase the microhardness and wear resistance of the original coating, the addition of SiC and TiC particles [22] can provide the coating with higher hardness and scratch resistance, and the addition of SiO_2_ [23,24,25] can change the structure of the coating, increase its nucleation points, increase the density of the coating, and provide it with better mechanical properties and corrosion resistance. With the deepening of research and as the preparation process matures, the performance of the coating can be further improved by adding more particles with different advantages, such as in Ni-P-Al_2_O_3_-PTFE [26], Ni-Cu-P-PTFE [27], and Ni-P-SiO_2_-Al_2_O_3_ [28], to adapt to more complex environments.

The above research shows that it is feasible to make coatings based on Ni-P that can be adapted to various environments by adding different particles. SiO_2_ is chemically stable, has a long service life and good economic value, and can also effectively improve corrosion resistance, while Cu displays good corrosion resistance and thermal stability. This study focuses on the low-temperature flue gas environment and aims to improve the corrosion resistance and antifouling performance of the heat exchanger pipeline at the dew point by applying a coating, thereby achieving the more efficient use of heat energy. Furthermore, since the price of high-performance steel is often several times that of ordinary steel, improving the performance of ordinary steel to reduce the use of high-performance steel will significantly lower the costs. In this study, Cu and SiO_2_ are added to a Ni-P coating to create a Ni-Cu-P-SiO_2_ coating, which can prevent or control corrosion and fouling in heat exchangers.

This study proposes a preparation process for a Ni-Cu-P-SiO_2_ coating based on a Ni-P coating. A well-dispersed, stable Ni-Cu-P-SiO_2_ coating can be prepared in a medium-temperature alkaline environment. The poor dispersion of SiO_2_ was overcome by replacing solid SiO_2_ particles with SiO_2_ in a gel state. The performance of the different coatings was characterized by means of electrochemical and immersion test methods to study the effect of the added particles on the performance of all coatings. To test the performance of the coatings in real low-temperature flue gas environments, a simulated flue gas heat transfer experimental platform was set up. Real flue gas environments were created by means of coal combustion to test the corrosion and fouling resistance of the coatings and to demonstrate the inhibitory effect of the coatings in the presence of acid and ash. Finally, recommendations are provided for selecting heat transfer materials for use in harsh waste heat recovery environments.

## 2. Experiments and Methods

### 2.1. Preparation Method of Coating

Heat exchange pipes made of Q235 steel, ND steel, or 316L steel and those coated with Ni-Cu-P, Ni-P-SiO_2_, and three types of Ni-Cu-P-SiO_2_ coatings were used in the experiments. The elemental compositions (except for Fe) of Q235 steel, ND steel, and 316L steel are shown in Table 1.

Ni-Cu (0.4 g/L)-P-, Ni-P-SiO_2_ (40 g/L)-, Ni-Cu (0.4 g/L)-P-SiO_2_ (20 g/L)-, Ni-Cu (0.4 g/L)-P-SiO_2_ (40 g/L), and Ni-Cu (0.4 g/L)-P-SiO_2_ (60 g/L)-coated tubes were prepared by means of electroless plating on a Q235 steel substrate. As Ni-P composite coatings are based on Ni, their physical properties, such as creep resistance, are similar to those of Ni layers.

The coating preparation steps were as follows: (1) Surface treatment: To improve the preparation process, a Q235 steel pipe was polished with 240, 400, 600, 800, and 1200 alumina sandpaper. The oxides, hydroxides, fouling, and unwanted deposits on the surface were removed by simple polishing, grinding, and brushing, so that the surface of the pipe to be plated can reach the required finish, i.e., the roughness (Ra) is lower than 5 μm. In industrial production, it is possible to prepare heat exchanger pipes with the same finish by mechanical means. (2) Alkali washing: The substrate after surface treatment was placed in the oil removal solution for alkali washing, and the oil and dirt on the surface of the substrate were removed via the saponification of the oil by the alkali solution. Before degreasing, the degreasing solution was heated to 70 °C, and the substrate was immersed in the degreasing solution for 8–10 min and washed with deionized water. (3) Electrolytic alkali washing: The surface of the pipe to be plated was used as the positive electrode of the DC power supply, while the 316L stainless steel plate of 5 cm × 10 cm was used as the negative electrode of the power supply. The positive and negative electrodes were immersed in the alkali washing solution at the same time, and the samples were electrolyzed at a voltage and current of 5V/3A. The oxygen precipitated from the surface of the material was used to further clean off the grease and dirt. The anode and cathode were not in contact with each other during cleaning, and the composition and operating conditions of the lye are shown in Table 2. (4) Pickling: After washing the matrix in the electrolytic alkali, the matrix was immersed in 15% hydrochloric acid at room temperature for 30–60 s. (5) Configuration of the plating solution and preparation of the coating: The main salt used was NiSO_4_, with NaH_2_PO_2_ serving as the reducing agent, and various additives were added to the plating solution. Methods for preparing different coatings are outlined in Table 3. Note that to ensure proper adhesion between the coating and the substrate, Ni-Cu-P and Ni-P-SiO_2_ composite coatings were prepared by means of a two-step method, that is, a Ni-P coating was first deposited on the surface of the substrate as the basis, and the Ni-P-SiO_2_ coating was prepared after the basic coating was deposited. When preparing Ni-Cu-P-SiO_2_ coating, the Ni-P coating was first deposited, the Ni-P-SiO_2_ coating was then deposited as the intermediate layer, and finally the Ni-Cu-P-SiO_2_ coating was deposited to obtain the target coating. The above reagents were all obtained from Shanghai McLean Company, and all drugs were analytical-grade reagents. (6) Heat treatment: The prepared samples were dried at 150–200 °C for 1 h. The chemical composition content of the coatings is shown in Table 4.

### 2.2. Experimental System

**Figure 1:**
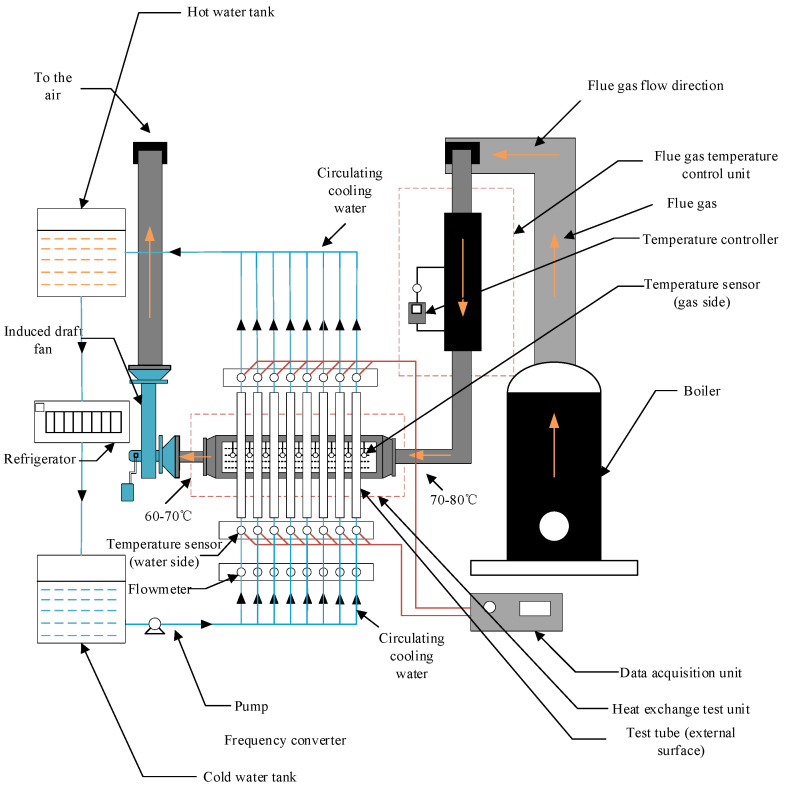
Schematic of the experimental system.

**Figure 2:**
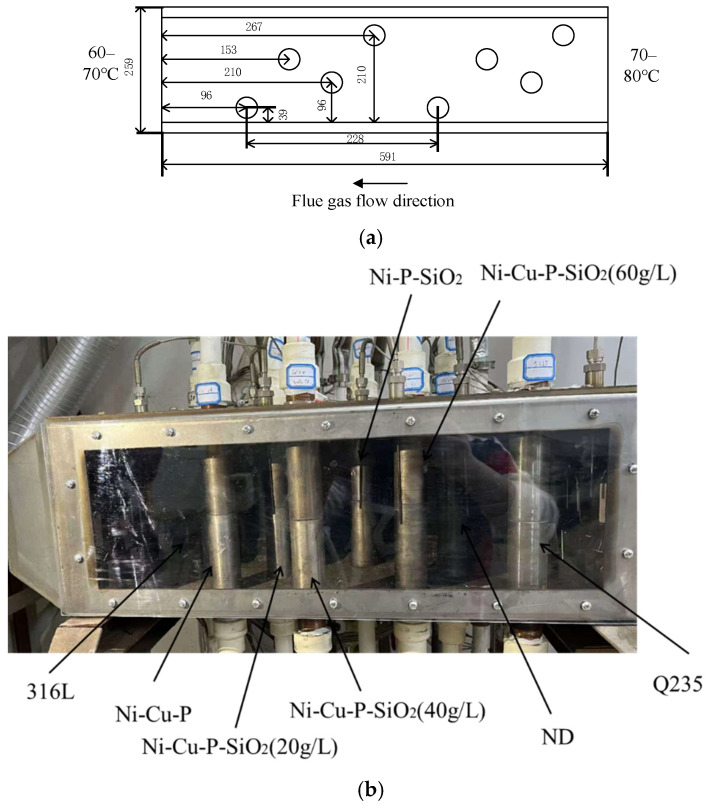
The heat transfer test unit. (**a**) Position of heat exchange tube bundle (mm); (**b**) structure of the test tube.

The experimental system for the coating test is illustrated in Figure 1. The system primarily comprises a coal-fired smoke supply unit, a heat transfer test unit, a flue gas circulation circuit, a water circulation circuit, and a data acquisition and processing unit. It is used to study the accumulation of ash and corrosion on the heat exchange surface of low-temperature flue gas in power plants, petrochemical plants, and so on. The main structure of the experimental device is a heat exchange test unit, which is used to simulate the heat exchange process in shell-and-tube heat exchangers. The temperature in the experimental device was controlled at 70–80 °C, and the temperature field changes according to the gradient law. The box’s front side adopts a visual transparent polycarbonate plate to facilitate the observation of the ash corrosion on the surface area of the test tube. This material can withstand a temperature of 350 °C and can provide an effective safety guarantee. The heat exchange tube bundle is staggered up and down, and the casing structure is adopted. The inner casing is a high-thermal-conductivity copper tube with an outer diameter of 32 mm and a length of 300 mm. The thread specification at both ends is M30 × 1.5, with a length of 50 mm. The test outer tubes are made of ND steel, Q235 steel, 316L stainless steel, and Ni-Cu-P, Ni-P-SiO_2_, Ni-Cu-P-SiO_2_ (20 g/L), Ni-Cu-P-SiO_2_ (40 g/L), and Ni-Cu-P-SiO_2_ (60 g/L)-coated pipes based on Q235. The pipe section has an outer diameter of 38 mm, a thickness of 3 mm, and a length of 10 mm. A total of two experimental pipe sections were set up. The specific structure is shown in Figure 2. At the same time, in order to improve the thermal conductivity, a layer of thermally conductive silicone grease is added between the inner tube and the outer tube. The cooling water flows in from the inner sleeve and flows out after heat exchange with the flue gas environment through the experimental pipeline. The heat transfer test unit is composed of the front side box and the rear side box, which are placed in parallel. The front side box is used for the heat transfer corrosion test, and the rear side box is placed with the same material for subsequent experimental exploration. Eight heat transfer tube bundles are placed in each box, and up to eight different materials can be used to conduct heat transfer experiments at the same time.

The flue gas circulation circuit is mainly composed of a flue and an induced draft fan. The flue is connected to the coal-fired smoke supply unit and thermal transfer test unit to ensure the smooth flow of the flue gas along the pipeline. The emissions from the boiler enter the heat exchange test unit through the flue for heat exchange and discharge. The flue gas temperature is controlled at 70–80 °C by a control device to verify the corrosion resistance of the coating in the case of dew point corrosion and to realize the possibility of maximizing the recovery of heat energy. At the same time, in order to prevent flue gas leakage, the connection part of each unit is wound with asbestos rope to strengthen the sealing. The water circulation circuit is composed of a cold-water tank, a hot-water tank, a refrigerator, an upper water tank, a lower water tank, a water pump, and a circulation pipeline. The water pump is used to provide power and regulate the flow of cooling water. In order to keep the cooling water temperature of the test tube constant, the refrigerator cools the water after heat transfer and discharges it into the cold-water tank to form a cycle. The resistance temperature sensor is used to measure the circulating water temperature at the inlet and outlet. The temperature sensors are set on the windward and leeward sides of each heat exchange tube to measure the flue gas temperature at the inlet and outlet. The anemometer and flowmeter are used to measure the flow rate of flue gas and water. Finally, the gathered data are captured by the data acquisition module and then sent to the computer for processing. The simulated flue gas heat transfer experimental device simulates the working environment of the flue gas heat exchanger in the power plant, so this experiment is closer to the real working condition.

The ultimate analysis of the coal used in the experiment after air drying is shown in Table 5. Among them, the elements N and S form oxides such as NO, NO_2_, SO_2_, and SO_3_ during combustion. When these oxides react with water and oxygen, they will generate acidic liquids like HNO_2_, HNO_3_, H_2_SO_3_, and H_2_SO_4_, causing corrosion.

The ash composition after coal combustion is shown in Table 6. These ashes will flow along with the flue gas and deposit on the surface of the heat exchange pipes, forming a loose and porous structure. This structure enhances the adsorption of corrosive media and exacerbates corrosion. The softening temperature of the ash is 1652 °C, while the combustion temperature of the boiler ranges from 1000 °C to 1300 °C. This paper focuses on researching a new type of coating suitable for deep recovery of low-temperature flue gas waste heat. The flue gas enters the heat exchanger after it is cooled to below 80 °C. Therefore, the ash flows along with the flue gas in the form of hard solids and is mainly deposited on the surface of the heat exchange pipes through collision and adhesion, so the softening temperature has little impact on the deposition of ash.

### 2.3. Experimental Process

During the operation of the experimental device, adjusting the flow rate of the water circulation cooling device of the atmospheric pressure boiler and the weight of the coal added each time can effectively adjust the inlet temperature. According to previous research on the characteristics of the flue gas ash scale and research by Chen et al. [11], when the wall temperature of the boiler is 70 °C, the products of the reaction of sulfides and nitrides in the flue gas with water and oxygen begin to adhere to the installed sensors, and it can be concluded that the acid dew point of sulfides should be higher than 70 °C. In the low-temperature flue gas heat exchanger, water droplets are likely to condense on the surface of the cooling water pipeline. NO_x_ and SO_x_ in the flue gas react with water and oxygen to form products such as H_2_SO_3_, H_2_SO_4_, HNO_2_, and HNO_3_, which corrode the pipelines. In the actual operation process, in order to shorten the experimental period and accelerate the formation of fouling and corrosion on the heat exchange surface, it was necessary to place the material in the most susceptible environment to fouling and corrosion, so the inlet flue gas temperature was set to 70–80 °C. Due to the size issue of the heat exchanger, every coated pipe is composed of two identical pipes connected in series. After 96 h of the experiment, one section is taken out for offline analysis. After the analysis is completed, it is restored to its original state to continue the experiment for another 96 h, with a total experimental cycle of 192 h. The removed heat exchange tube bundle was brushed with a brush to remove the surface area ash and corrosion products and properly preserved, and the cross-section of the heat exchange tube bundle was obtained for coating analysis. An FEI Versa3D field emission scanning electron microscope (Thermo Fisher Scientific, Waltham, MA, USA) was utilized to investigate the microstructure, structural characteristics, surface ash, and corrosion product composition of the eight different materials. The elemental composition of the coating was analyzed using an energy-dispersive spectrometer (EDS), and the repeatability of the preparation of the coating was confirmed. The specific material and crystal structure of the coating surface were obtained by an X-ray diffractometer (XRD). Equipment parameters: Under 45 kV and 200 mA Cu Kα radiation, the scanning rate was 4°/min, and the phase composition of the coating was determined.

Electrochemical testing was carried out with the CHI660E workstation from Shanghai Chenhua Instrument Company (Shanghai, China). Since the corrosion of the working environment of the heat exchange tube bundle was dominated by sulfur oxides, 5% sulfuric acid [29] solution was used as the electrochemical solution to analyze the corrosion resistance characteristics of different materials in the same environment, and the above dynamic implementation was mutually verified to ensure the authenticity of the experimental results. The experiment was conducted at room temperature utilizing a standard three-electrode system, with a saturated calomel electrode (SCE) as the reference, a platinum (Pt) sheet as the auxiliary electrode, and the sample to be tested as the working electrode. When the electrochemical workstation was working, the test sample was placed in 5% sulfuric acid solution, and only a surface area of 1 cm × 1 cm was exposed. After standing for one hour, the polarization curve and impedance spectrum of the material were measured after the open circuit potential became stable. To ensure the accuracy of the final results, the results for each sample were averaged over three parallel experiments. The polarization curve test’s potential scanning speed was 0.5 mV/s, with electrochemical impedance spectroscopy using open circuit potential measurement. The amplitude of the AC signal was 5 mV, the frequency range was 100,000~0.01 Hz, and the scanning direction was from high frequency to low frequency.

In addition, samples of various materials were taken and immersed in 5% sulfuric acid solution for 168 h continuously. The samples were taken out every 24 h, and the impurities on the surface were washed away with running water. The corrosion products on the surface were gently brushed off with a brush, and the weight change in the sample was measured.

### 2.4. Analysis Method

For the flue gas heat exchanger, the temperature set in the experiment is relatively low and all the experimental pipes have been polished, which means the emissivity is low, so the convective heat transferring dominates. The efficiency loss of the heat exchanger is low due to the use of thermal insulation measures. Therefore, it is approximated that the heat absorbed by the cooling water should be equal to the heat released by the flue gas so that dynamic equilibrium is achieved, and calculating the average of heat absorption and heat release as the heat exchange quantity can reduce the error caused by heat exchange efficiency loss.

(1) $$Q_{w}=Q_{g}$$

(2) $$Q_{w}=\rho_{w}q_{w}C_{w}(t_{2}-t_{1})$$

(3) $$Q_{g}=\rho_{g}U_{g}SC_{g}(T_{1}-T_{2})$$

Here, $Q_{w}$ represents the quantity of heat absorbed by the cooling water, $Q_{g}$ is the quantity of heat released by the flue gas, $\rho_{w}$ is the density of the cooling water, $\rho_{g}$ is the density of the flue gas, $q_{w}$ is the volumetric flow rate of the water, which is the measurement value obtained directly with the flow meter, $C_{w}$ is the specific heat capacity of the cooling water, and *t*_1_ and *t*_2_ are the inlet and outlet water temperatures, respectively. $U_{g}$ is the flow velocity of flue gas, which is the measurement value obtained directly from the gas flow meter. *S* is the cross-sectional area of the heat exchanger, and $C_{g}$ is the specific heat capacity of flue gas.

(4) $$Q_{a}=\frac{Q_{w}+Q_{g}}{2}$$

Here, $Q_{a}$ is the heat transfer quantity of different materials between flue gas and cooling water; the heat transfer efficiency error is further reduced by averaging.

(5) $$K=\frac{Q_{a}}{A_{0}\Delta T_{m}}$$

Here, *K* is the heat transfer coefficient of the material, *A*_0_ is the effective heat transfer area, and $\Delta T_{m}$ is the logarithmic mean temperature difference.

(6) $$k_{0}=\frac{1}{\frac{1}{h_{g}}+\frac{\delta_{wall}}{\lambda_{wall}}\ln\frac{d_{0}}{d_{i}}+\frac{1}{h_{w}}\frac{d_{i}}{d_{0}}}$$

Here, *k*_0_ is the initial heat transfer coefficient, 1/*h_w_* is the thermal resistance of the cooling water side, *h_w_* is the convective heat transfer coefficient of the water side, 1/*h_g_* is the thermal resistance of the flue gas side, and *h_g_* is the convective heat transfer coefficient of the flue gas side. *δ_wall_* and *λ_wall_* are the wall thickness and thermal conductivity, respectively. *d*_0_/*d_i_* represents the proportion between the outer diameter and the inner diameter of the heat exchange tube.

(7) $$k=\frac{1}{\frac{1}{h_{g}}+\frac{\delta_{wall}}{\lambda_{wall}}\ln\frac{d_{0}}{d_{i}}+\frac{1}{h_{w}}\frac{d_{i}}{d_{0}}+\frac{\delta_{f}}{\lambda_{f}}}=\frac{1}{\frac{1}{k_{0}}+R_{f}}$$

Here, *k* is the heat transfer coefficient after fouling, $\delta_{f}$ and $\lambda_{f}$ are the thickness and thermal conductivity of the fouling layer, respectively, and $R_{f}$ is the fouling resistance, indicating the degree of fouling.

(8) $$R_{f}=\frac{1}{k}-\frac{1}{k_{0}}$$

We define the weakening degree of heat transfer coefficient ϕ as follows:

(9) $$\phi =\frac{k_{0}-k}{k_{0}}$$

## 3. Results and Discussion

### 3.1. Characterization of Morphology and Composition

**Figure 3:**
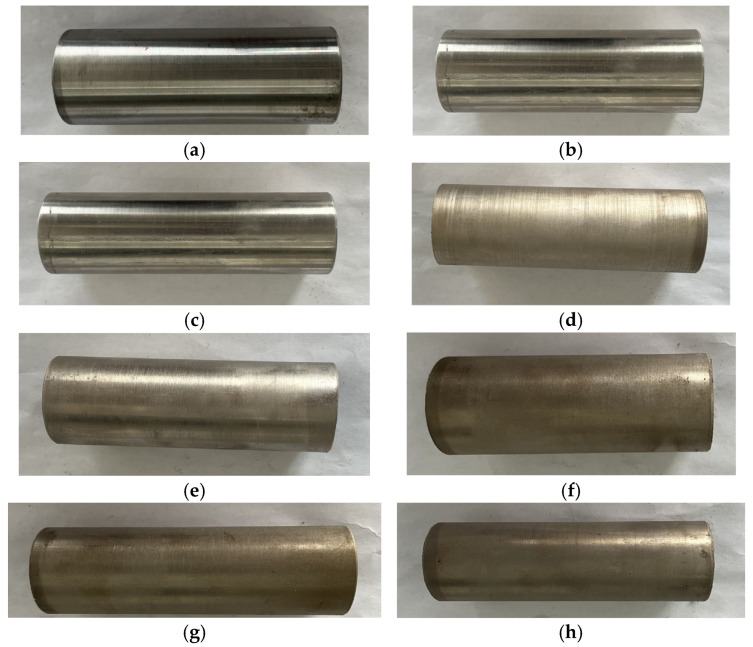
Test samples: (**a**) 316L; (**b**) ND; (**c**) Q235; (**d**) Ni-Cu-P; (**e**) Ni-P-SiO_2_; (**f**) Ni-Cu-P-SiO_2_ (20 g/L); (**g**) Ni-Cu-P-SiO_2_ (40 g/L); (**h**) Ni-Cu-P-SiO_2_ (60 g/L).

The visual aspect of the experimental pipes is shown in Figure 3. The outer diameter of the pipe section is 38 mm, the thickness is 3 mm, the length is 10 mm, and the roughness Ra ≤ 5 μm at the initial stage of the experiment. There are no significant differences between the three substrates, all of which are bright silver, after mechanical polishing. The Ni-Cu-P coating has a bright surface, with low roughness and no obvious cracks or holes. Compared with Ni-Cu-P, Ni-P-SiO_2_ has greater roughness and a dark surface. There were no obvious differences between the three different concentrations of Ni-Cu-P-SiO_2_ coatings, and the surface showed a brass color. With the increase in SiO_2_ concentration, the surface color deepened. There was no delamination and peeling on the surface of all coatings, and no self-reaction occurred during the preparation process.

**Figure 4:**
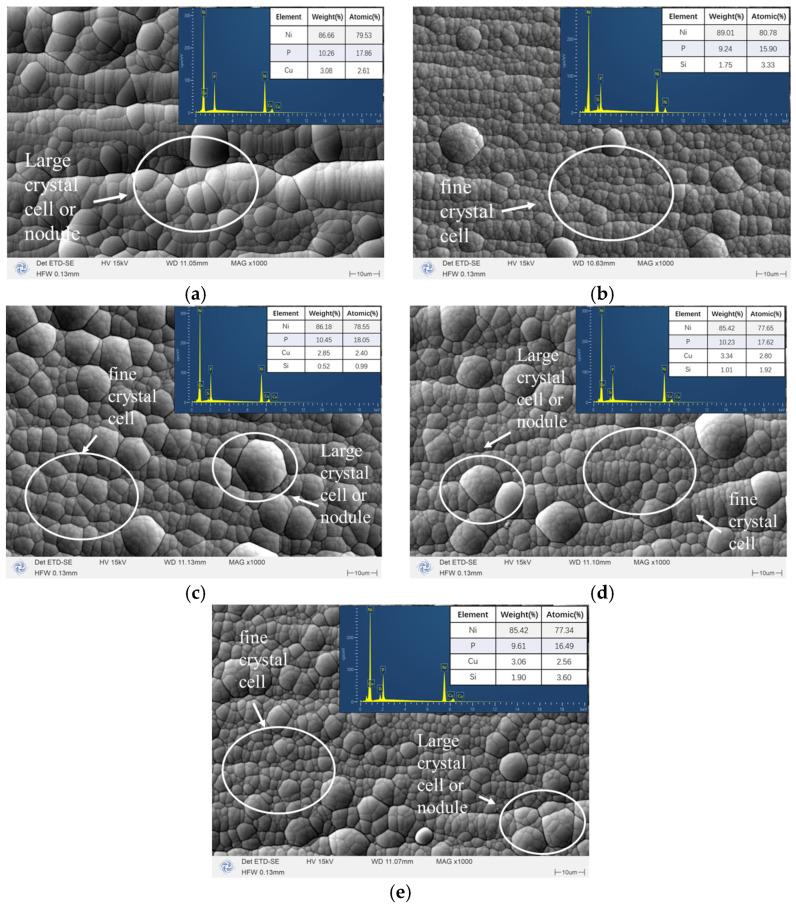
SEM and EDS images of each surface material: (**a**) Ni-Cu-P; (**b**) Ni-P-SiO_2_; (**c**) Ni-Cu-P-SiO_2_ (20 g/L); (**d**) Ni-Cu-P-SiO_2_ (40 g/L); (**e**) Ni-Cu-P-SiO_2_ (60 g/L).

The surface morphology and elemental contents of the coatings are shown in Figure 4. It can be seen from this figure that the coating formed by adding other elements based on Ni-P presents a typical continuous cauliflower structure, but due to the different types of added particles, the coating forms slight differences. By comparison, it was found that the surface of the Ni-Cu-P coating was uniform and smooth, with a large number of circular nodules. The controllable introduction of Cu ions activated the natural nucleation sites, resulting in more nuclear production [30], accelerated deposition, and the generation of large crystal cells. The Ni-P-SiO_2_ coating presents an island-like growth structure under the microscope, in which there are large spherical particles and a fine grain structure. Compared with the Ni-Cu-P coating, this coating shows obvious refinement characteristics [23]. The deposition of SiO_2_ creates a high density of nucleation sites and hinders crystal cell enlargement by not participating in the growth process of the coating crystals. As a result, the cellular structure is finer, and the geometry of the structure is smaller. The three Ni-Cu-P-SiO_2_ coatings display little difference, and the morphology contains the characteristics of the above two kinds of particles, that is, large circular nodules are produced, and there are obvious refinement characteristics. The large, round nodules are connected to each other by a dense, cell-like structure and have the typical characteristics of both particles, affecting crystal growth. The P content of the coatings is 9–10%. This level of P tends to form an amorphous structure, which gives the coatings better corrosion resistance. The content of SiO_2_ in the Ni-Cu-P-SiO_2_ coatings with three different concentrations was 0.99%, 1.92%, and 3.60%, respectively, indicating that the plating solution formula was controllable and reproducible, enabling the preparation of coatings with varying SiO_2_ concentrations.

**Figure 5:**
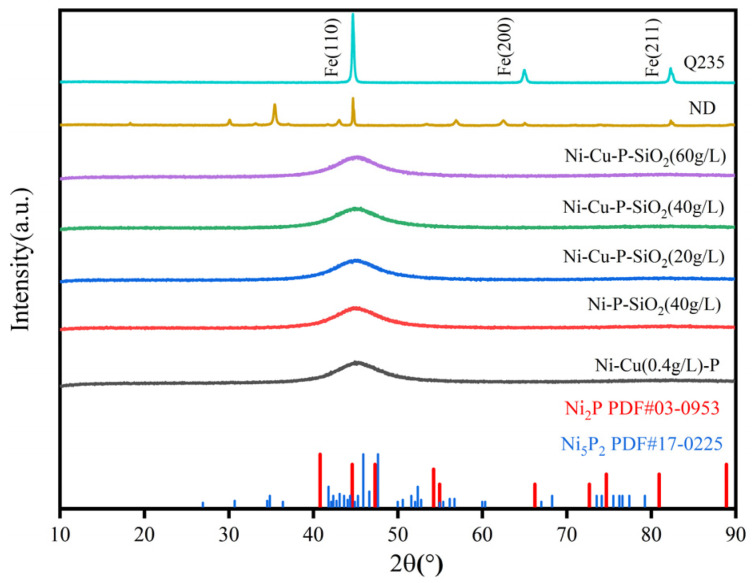
XRD images of each surface material.

The XRD method was used to determine the phase composition of the substrate and the coatings, and the findings are displayed in Figure 5. It can be seen that the ND steel and Q235 matrix are composed of crystals, and the X-ray diffraction peak is 44.9° at 2θ, which is exactly consistent with the iron plane (110) (45.1°at 2θ, JCPDS No. 01-1267). The remaining coating surfaces are in an amorphous state, displaying very broad diffraction peaks. This indicates that the Ni-P coatings are not well crystallized, the major elements are present in the amorphous forms of Ni_2_P (PDF: 03-0953) and Ni_5_P_2_ (PDF: 17-0225), and that the doping of the nanoparticles has no effect on the phase transition.

### 3.2. Antifouling Performance Analysis

**Figure 6:**
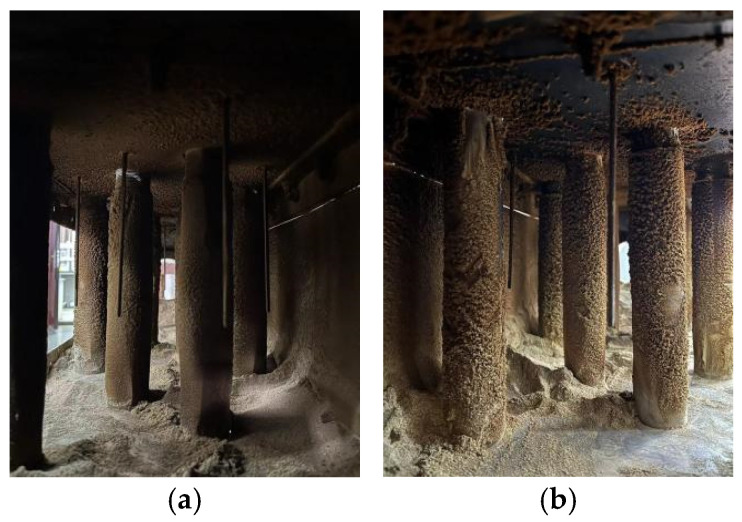
Fouling of heat exchange test unit: (**a**) before fouling; (**b**) after fouling.

Because the ash deposition state and condition of the windward and leeward sides of the heat transfer test unit are different, photos of the heat transfer test unit after the experiment were taken, as shown in Figure 6. In this figure, Figure 6a is the windward side state, and Figure 6b is the ash deposition state of the leeward side. Before the start of the experiment, the surface of the heat exchange tube bundle is bright. When the experiment is over, a thick layer of dirt is condensed on the surface of the heat exchange tube, and the amount of dirt on the windward side is great, the density is high, and the whole surface is covered. The fouling on the leeward side is relatively minimal and unevenly distributed, forming many small ash masses. This is due to the different deposition modes of dust on the windward and leeward sides. At lower wall temperatures and lower wind speeds, the dust is solid, spherical, and less affected by temperature. Inertia settlement mainly occurs on the windward side, that is, the airflow carries ash particles and collides with the heat exchange tube bundle. When the adhesion force is greater than the inertia force, the dust will adhere to the heat exchange tube bundle and gradually grow and form a loose state [31]. The dust on the leeward side mainly undergoes vortex deposition, and the characteristics of circuitous flow are formed during the gas flow process. In this process, dust consisting of small particles adheres to the surface after contact with the heat exchange tube bundle to form fouling. At the same time, due to the impact–adhesion mechanism, most of the small dust particles adhering to the surface for the first time are blown away by the air flow as the experiment progresses, which is also the reason why the amount of leeward dust is lower than that for the windward side.

After the experiment is completed, the heat exchange tube bundle is removed, and the ash deposition on the tube bundle is processed. It was found that the dust has a clear layered structure. Fine ash forms a thin initial ash layer on the innermost side, and then the outermost side comprises coarse ash. During the experiment, the outermost ash will periodically peel off, and the same phenomenon is observed on the surface of the temperature sensor next to the heat exchange tube bundle, which corresponds to the ash deposition characteristics studied by Zhang et al. [32].

**Figure 7:**
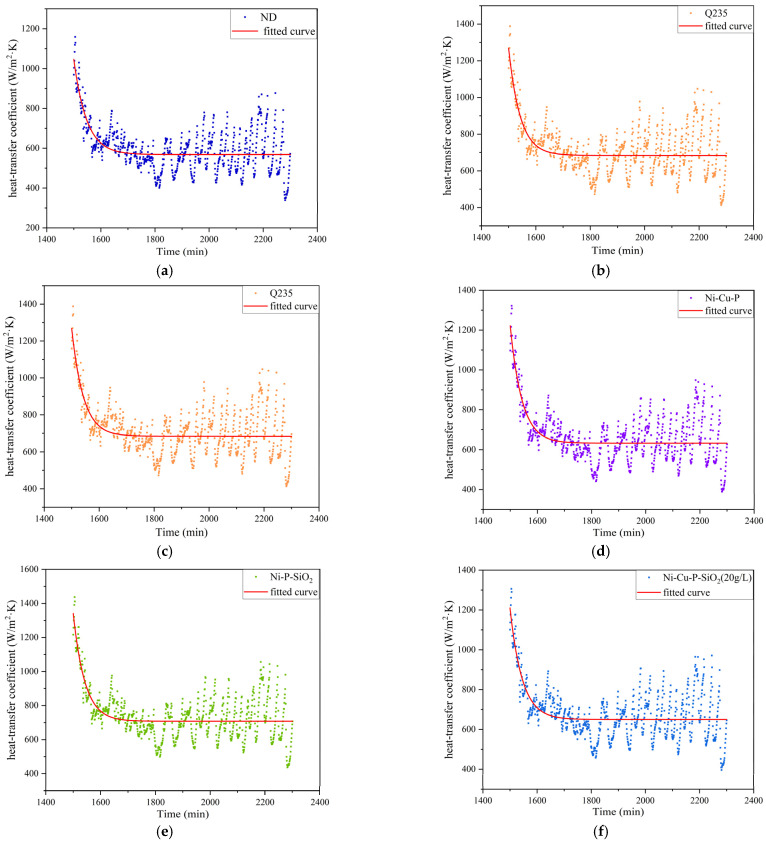

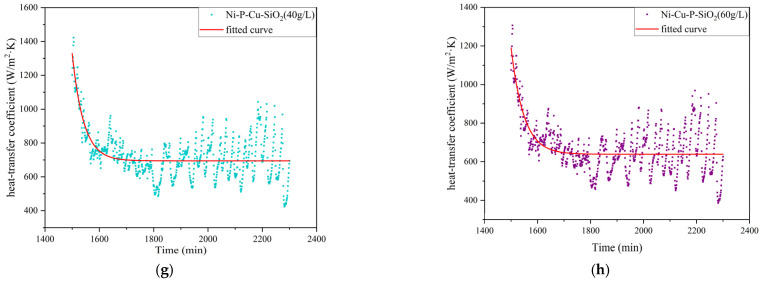
Results of monitoring the heat transfer coefficient for each surface material: (**a**) ND; (**b**) Q235; (**c**) 316L; (**d**) Ni-Cu-P; (**e**) Ni-P-SiO_2_; (**f**) Ni-Cu-P-SiO_2_ (20 g/L); (**g**) Ni-Cu-P-SiO_2_ (40 g/L); (**h**) Ni-Cu-P-SiO_2_ (60 g/L).

Meanwhile, the system evaluates the heat exchange performance by calculating the heat transfer coefficient based on these data such as inlet and outlet flue gas temperatures and water temperatures, monitoring and recording in real time. Because the operation of the system is affected by many factors, the data obtained under the initial conditions are inaccurate. After the system becomes stable, the experimental data are recorded, and curve fitting is performed. The results are shown in Figure 7.

The collected data fluctuate due to the instrument, measurement, and calculation process used, but the overall trend is consistent, which decreases first and then becomes stable. This corresponds to the study by Chen et al. [11]. In the case of a low wall temperature, the deposition of ash is aggressive and will affect the heat transfer coefficient. After the amount of ash accumulation reaches a certain level, the total amount of ash accumulation reaches a dynamic equilibrium, and the heat transfer coefficient becomes stable.

**Figure 8:**
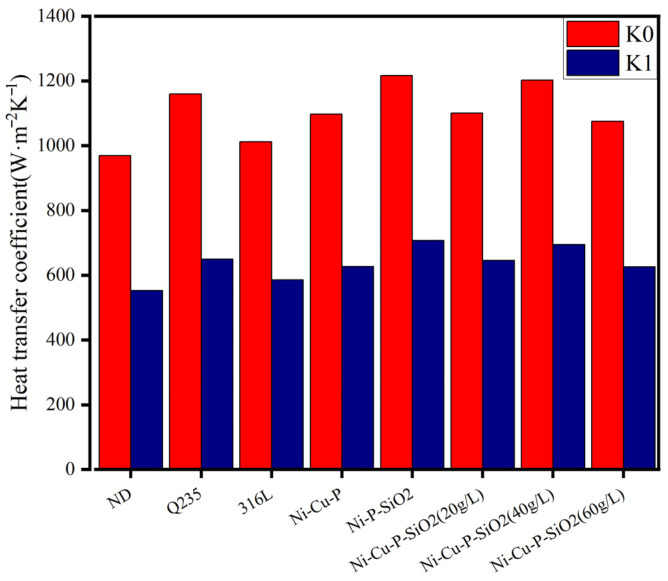
Results comparing the heat transfer coefficients for each surface material before and after fouling.

To compare the heat transfer performance of various materials, the initial value of the fitting curve is designated as K0, representing the heat transfer coefficient of the clean tube bundle. The stable final value is designated as K1, representing the heat transfer coefficient of the dirt. K0 and K1 are shown in Figure 8. It can be seen from Figure 8 that the K1 values of various materials are ranked as Ni-P-SiO_2_ > Ni-Cu-P-SiO_2_ (40 g/L) > Ni-Cu-P-SiO_2_ (20 g/L) > Q235 > Ni-Cu-P-SiO_2_ (60 g/L) > Ni-Cu-P > 316L > ND. From this overall trend, the heat transfer coefficient of the coated tubes is seen to be greater than the heat transfer coefficient of the three matrix materials.

**Figure 9:**
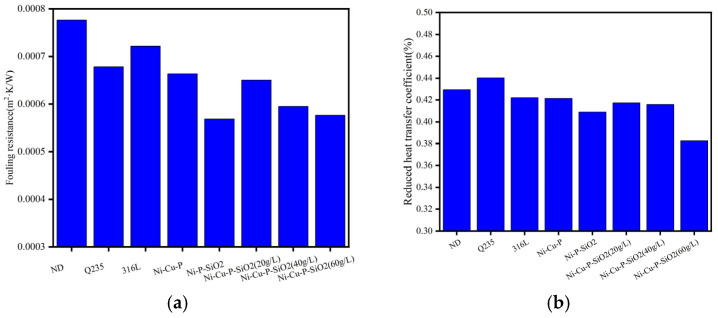
Comparison results of fouling resistance and reduced heat transfer coefficient: (**a**) fouling resistance (Rfo); (**b**) weakened heat transfer coefficient (φ).

The reason why Q235 has a higher heat transfer coefficient may be that in the whole system structure design, Q235 is located closest to the inlet flue gas temperature side, so it has a higher flue gas temperature and thus a higher heat transfer coefficient. In order to more accurately express the influence of the coating on the antifouling performance, the fouling thermal resistance (Rfo) and the weakening coefficient (ψ) of various materials before and after the test were calculated, as shown in Figure 9.

According to the overall trend analysis of the fouling resistance and weakening heat transfer coefficient, the ranking of Rfo and ψ for various materials from small to large is Ni-Cu-P-SiO_2_ (60 g/L) > Ni-P-SiO_2_ > Ni-Cu-P-SiO_2_ (40 g/L) > Ni-Cu-P-SiO_2_ (20 g/L) > Ni-Cu-P > 316L > ND > Q235. The overall trend of the fouling resistance and weakening heat transfer coefficient is consistent. Compared with the above results, this shows that the coating has good fouling resistance.

### 3.3. Analysis of Corrosion Resistance

#### 3.3.1. Electrochemical Test

**Figure 10:**
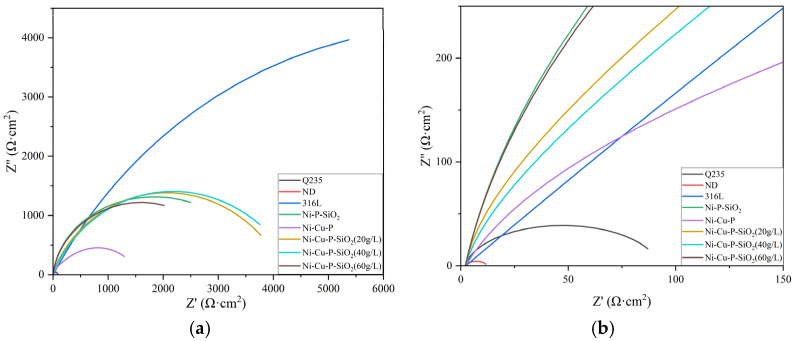
The Nyquist plot of each surface material. (**b**) is a partial enlargement of (**a**).

Electrochemical impedance spectroscopy can reveal the electrochemical change process of the surface coatings of materials in a solution, thereby studying the corrosion resistance of carbon steel, stainless steel, and different coating materials. Figure 10 shows the electrochemical impedance Nyquist plots of several materials in 5% H_2_SO_4_ solution, where (b) is a partial enlarged view of (a). The coatings have a protective effect on the substrate sample. A dense and stable coating can prevent the sample from directly contacting and reacting with the test solution, thereby slowing down the corrosion process. It can be seen from the changes in the impedance spectrum that the Nyquist plots of all eight materials in the sulfuric acid solution each consist of a capacitive reactance arc. Among them, the 316L stainless-steel sample has the largest radius of the capacitive reactance arc, while the Q235 steel and ND samples have smaller radii of the capacitive reactance arc, indicating that the carbon steel samples have lower resistance in the test solution. Compared with carbon steel samples, the radii of the capacitive reactance arcs of the two coated samples (Ni-Cu-P and Ni-P-SiO_2_) with Q235 steel as the substrate increase, indicating that the corrosion resistance of the materials in the solution has been improved. Moreover, the three Ni-Cu-P-SiO_2_-coated samples with different additive amounts of coating components have larger capacitive reactance arcs, showing excellent corrosion resistance. Among them, when the addition amount of SiO_2_ sol is 40 g/L, the capacitive reactance arc is the largest.

**Figure 11:**
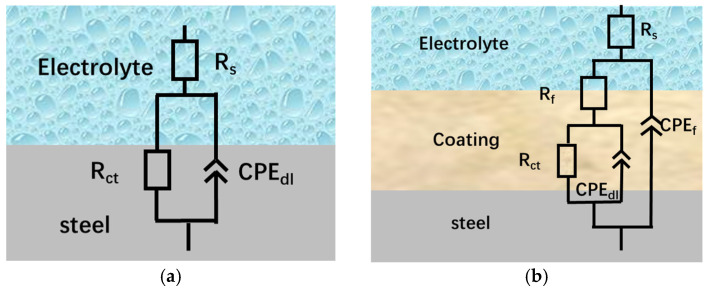
Equivalent circuit of electrochemistry: (**a**) for uncoated tubes; (**b**) for coated tubes.

The AC impedance data were fitted by the equivalent circuit shown in Figure 11. The fitting results are presented in Table 7, and the percentage errors of the fitting data are shown in Table 8. In the equivalent circuit, R_s_, R_f_, and R_ct_ represent the solution resistance, coating film resistance, and electrochemical corrosion reaction resistance (also known as charge transfer resistance), respectively. R_f_ is related to the structure and porosity of the coating. When electrochemical corrosion reactions occur through the electric double layer on the material surface via charges (electrons and electrolyte ions), there may be resistance to charge transfer, which is the electrochemical reaction resistance R_ct_ in the solution. In general, the larger the R_ct_, the more difficult it is for the electrochemical reactions that cause corrosion to proceed and thus the better the corrosion resistance [33]. The corrosion protection can often be reflected by an increase in R_ct_, as a higher R_ct_ value indicates that the coating or inhibitor has successfully reduced the corrosion rate.

It can be seen from the fitting parameters in Table 7 that the charge transfer resistance (R_ct_) of the ND sample is low at 9.641 Ω·cm^2^, followed by that of Q235 steel, which is 90.97 Ω·cm^2^. The low R_ct_ of these materials indicates that charge transferring at the interface is easier, resulting in a faster corrosion rate for the materials. On the exposed metal surface, the charge transferring resistance is usually low, and the corrosion process is active. 316L stainless steel contains a high amount of Cr, Ni elements relatively and a small amount of Mo (molybdenum). The Cr element can form a dense Cr_2_O_3_ oxide layer on the surface, preventing the further occurrence of corrosion reactions [34]. Ni can improve the corrosion resistance of the steel, especially in acidic environments, reducing the risk of intergranular corrosion [35]. Therefore, 316L stainless steel has the largest resistance value in 5% H_2_SO_4_ solution, which is 14,357 Ω·cm^2^. The R_ct_ values of the five coated samples in descending order are as follows: 4130 Ω·cm^2^ (Ni-Cu-P-SiO_2_ (40 g/L)) > 3918 Ω·cm^2^ (Ni-Cu-P-SiO_2_ (20 g/L)) > 1710 Ω·cm^2^ (Ni-P-SiO_2_) > 1324 Ω·cm^2^ (Ni-Cu-P-SiO_2_ (60 g/L)) > 1112 Ω·cm^2^ (Ni-Cu-P). Through comparative analysis, it can be found that the R_ct_ values of the materials with coatings are all increased, indicating that the charge transfer process is inhibited.

Coating film resistance is an important indicator of the compactness and uniformity of a coating. A larger R_f_ value indicates better compactness of the coating, which can effectively block corrosive media; a smaller R_f_ value, on the other hand, suggests that the coating may have pores or defects, reducing its barrier effect. In EIS testing, a higher coating film resistance value usually means that the coating is uniform and dense, which can provide excellent barrier performance and delay the occurrence of corrosion reactions [36].

The addition of copper significantly refines the grains, improves the compactness of the coating, reduces the grain size and porosity of the coating, and makes the surface of the Ni-Cu-P coating flat. However, the doping of copper will alter the integrity of the grains, thereby reducing the corrosion resistance and decreasing the coating film resistance. As a physical barrier, SiO_2_ nanoparticles can fill the micropores in the coating, block the penetration path of corrosive media, and at the same time enhance the compactness of the coating. Due to the insulating property of SiO_2_, it can prevent the occurrence of galvanic corrosion, which further improves the corrosion resistance of the Ni-P-SiO_2_ coating and increases the coating film resistance. Nevertheless, SiO_2_ has certain limitations. If SiO_2_ is unevenly dispersed or weakly bonded to the metal matrix, particle detachment may occur in high-temperature or acidic environments, leading to local pitting corrosion.

The incorporation of Cu nanoparticles leads to the formation of the Ni_3.8_Cu phase in the coating after heat treatment [37], which optimizes the metallic structure. The Ni-Cu-P passive film with bipolar semiconductor properties can not only prevent the inflow of anions but also restrict the outflow of cations, which enhances the protective ability and corrosion resistance of the Ni-Cu-P passive film and promotes the formation of the passive film [38]. P and SiO_2_ in the coating act as a barrier layer on the substrate surface [39]. Experiments show that the self-corrosion current density of the Ni-Cu-P-SiO_2_ coatings is lower than that of the Ni-Cu-P coating, and the impedance value is higher. Among them, the charge transfer resistance of the Ni-Cu-P-SiO_2_ (40 g/L) sample is relatively large, which is four times that of the Ni-Cu-P coating.

The value of Y_0_ is closely correlated with the capacitive properties of the electrode material, the effective surface area of the electrode, and the electrolyte concentration in the solution. In corrosion studies, Y_0_ denotes the pore density on the electrode surface [40], thus reflecting the quality of the coating. A higher Y_0_ may imply that there are more pores (i.e., corrosion active sites) on the electrode surface, and the corrosion reaction is more vigorous, while a lower Y_0_ usually means that the surface is covered with corrosion products or a passive film, which reduces the capacitive characteristics of the electric double layer. By comparing the fitting parameters, it can be found that ND has a relatively high Y_0_ value, indicating that there are more active sites on the surface of the sample, making it more prone to electrochemical corrosion. Secondly, among the coated samples, Ni-Cu-P and Ni-P-SiO_2_ also have higher Y_0_ values, so they have a greater possibility of chemical corrosion. The three types of Ni-Cu-P-SiO_2_ coatings have lower Y_0_ values, among which the Ni-Cu-P-SiO_2_ (20 g/L) coating has the smallest value, indicating that the coating of this sample is more uniform and denser. The Y_0_ value of the Ni-Cu-P-SiO_2_ (40 g/L) coating is second only to that of the Ni-Cu-P-SiO_2_ (20 g/L) coating, and it can still maintain sufficient corrosion resistance.

The measurement of polarization curves is conducted in the strong polarization region, where the potential (E) is very large (usually greater than 100 mV). In the strong polarization region (Tafel region) of the polarization curve, the applied current and electrode polarization follow the Tafel relationship. From the intersection of Tafel lines, electrochemical parameters such as the corrosion potential (E_corr_) and corrosion current (I_corr_) of the corroding metal electrode can be derived, so as to evaluate the corrosion resistance of the material. The strong polarization region testing method is one of the most classical electrochemical testing methods for corrosion rates.

**Figure 12:**
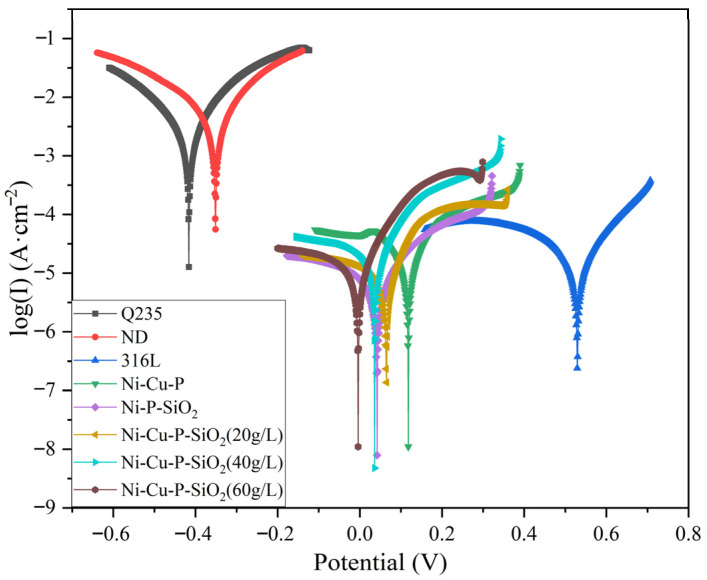
Polarization curves of each surface material.

Potentiodynamic polarization scanning was performed on eight groups of specimens, and the electrochemical parameters including self-corrosion potential (E_corr_) and self-corrosion current density (I_corr_) were obtained by Tafel line extrapolation. Figure 12 shows the polarization curves of several specimens in 5% H_2_SO_4_ solution. All materials were measured three times, and the mean value and standard deviation were calculated. The electrochemical results corresponding to the polarization curves are shown in Table 9. The 316L stainless steel specimen has a relatively high corrosion potential in the test solution, indicating that 316L stainless steel has a low corrosion tendency. ND and Q235 steel specimens showed low corrosion potential values and large current values, suggesting that those specimens have a fast corrosion rate in H_2_SO_4_ solution. The current of the five coated specimens decreases, and the corrosion potential of the materials in the solution shifts positively, indicating that the corrosion rate of the specimens decreases and the corrosion resistance of the materials is improved [18].

It can be found from the corresponding fitting parameters that Q235 and ND steels have high self-corrosion currents (I_corr_), which are 3.562 × 10^−3^ A·cm^−2^ and 5.776 × 10^−3^ A·cm^−2^, respectively, and their self-corrosion potentials (E_corr_) are −0.343 V and −0.424 V, respectively. Compared with Q235 and ND steels, the corrosion potential of the Ni-Cu-P-coated material shifts positively to 0.106 V, and the Icorr decreases to 4.220 × 10^−5^ A·cm^−2^; the I_corr_ of the Ni-P-SiO_2_ specimen decreases to 1.038 × 10^−5^ A·cm^−2^.

According to electrochemical theory, the corrosion current density (I_corr_) of a metal electrode is proportional to the corrosion rate, which can reflect the speed of metal corrosion. A higher I_corr_ value indicates a faster corrosion rate of the sample in sulfuric acid solution. Therefore, the Ni-P-SiO_2_ coating is superior to the Ni-Cu-P coating, and the Ni-Cu-P-SiO_2_ coating with a synergistic effect exhibits optimal performance. Among them, the self-corrosion potentials of the two coating samples, namely Ni-Cu-P-SiO_2_ (20 g/L) and Ni-Cu-P-SiO_2_ (40 g/L), are both above 0.03 V, and their self-corrosion current densities (I_corr_) are both lower than 2.3 × 10^−5^ A·cm^−2^, indicating that the materials have good corrosion resistance. Although the Ni-P-SiO_2_ coating has the lowest self-corrosion current level among the coating samples under sulfuric acid solution conditions, considering the results of charge transfer resistance and capacitive reactance arc, the Ni-Cu-P-SiO_2_ (40 g/L) coating has the best corrosion resistance.

#### 3.3.2. Soaking Experiment

**Figure 13:**
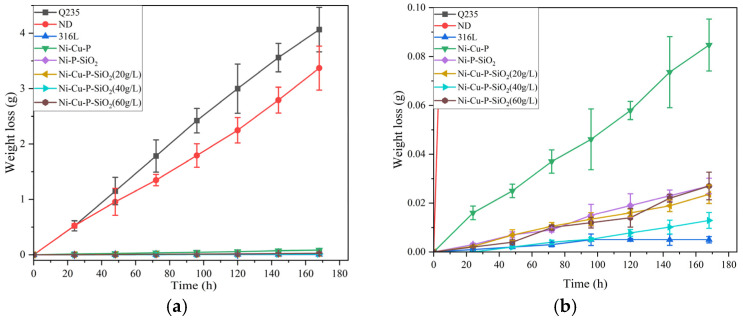
The weight loss of each surface material. (**b**) is a partial enlargement of (**a**).

The following figure presents the corrosion weight loss of the matrix. Each material was tested three times and was measured once every 24 h and then averaged. The results are shown in Figure 13.

**Figure 14:**
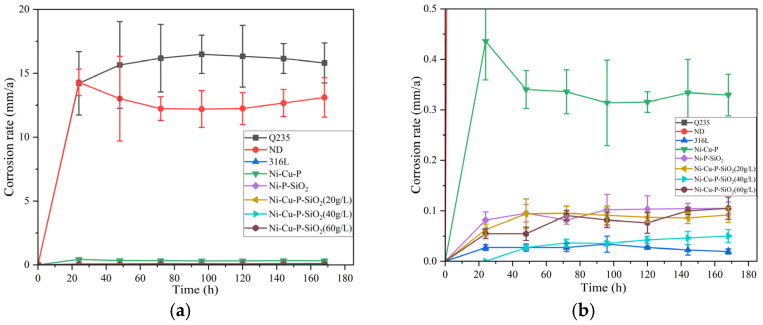
The corrosion rate of each surface material. (**b**) is a partial enlargement of (**a**).

According to the national standard GB/T 39534-2020 [41], the formula for calculating the corrosion rate is shown in Equation (10), where ν is the corrosion rate of the material, Δm is the mass difference before and after corrosion (g), *S* is the total area of the pattern (mm^2^), $t^{\prime }$ is the immersion time (h), and $\rho^{\prime }$ is the material density (g/mm^3^). The corrosion rate results are shown in Figure 14, and the average corrosion rate and standard deviation are shown in Table 10.

(10) $$\nu =\frac{8760\times \Delta m}{S\times \rho^{\prime }\times t^{\prime }}$$

The average corrosion rate of Q235 was the highest, being 15.83 mm/a during the experimental period, and the average corrosion rate of ND steel was slightly weaker than that of Q235, at 12.82 mm/a. The corrosion rates of the coating samples were all much lower than those of ND and Q235, among which the lowest corrosion rate of the Ni-Cu-P-SiO_2_ (40 g/L) coating was 0.03 mm/a. Therefore, the corrosion resistance of all materials is as follows: 316L = Ni-Cu-P-SiO_2_ (40 g/L) > Ni-Cu-P-SiO_2_ (60 g/L) > Ni-Cu-P-SiO_2_ (20 g/L) > Ni-P-SiO_2_ > Ni-Cu-P > ND > Q235. This is in agreement with the results of the electrochemical tests. However, the corrosion product coverage meant that the corrosion rate measurements were not entirely consistent with the electrochemical tests. Nevertheless, it was proven that the coating offered the best corrosion resistance when 0.4 g/L of CuSO_4_ and 40 g/L of SiO_2_ colloid were added.

#### 3.3.3. Analysis of Corrosion Resistance Performance in Low-Temperature Flue Gas Environment

After the completion of the aforementioned experiment, EDS line scanning technology was employed to scan the cross-section of the heat exchange tube in order to investigate its corrosion condition. Since the heat exchange tube bundle only comes into contact with the flue gas on the outer surface and forms corrosion products and deposits, this study only analyzes the outer surface of the test tube, and the cross-sectional scanning electron microscope cross-section of each surface material after corrosion is shown in the diagram. The black area on the right side of the figure is the resin used for mosaic when shooting the SEM image, and the black area on the left side is the matrix material.

SEM images show that the boundaries between coatings and between the coating and the substrate are clear, with good adhesion. There are no pores, the coating is evenly distributed, and the thickness is the same. During the EDS line scan, the point of significant variation in element concentration marks the boundary between the substrate and the coating, and at the junction of different coatings, the element content fluctuates significantly, and the boundaries between the coatings are clear. For the five coatings, very clean surfaces can be seen without visible Cu particles and SiO_2_ particles, possibly due to their smaller size and relatively low content. The layered structure of the coating has been marked in the figure, and the thickness of the Ni-P and Ni-P-SiO_2_ coatings as the transition layer is significantly smaller than that of the outermost layer.

**Figure 15:**
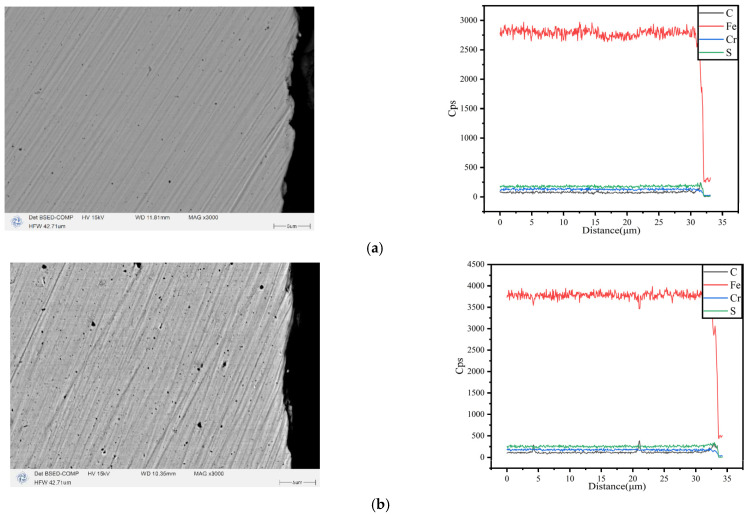

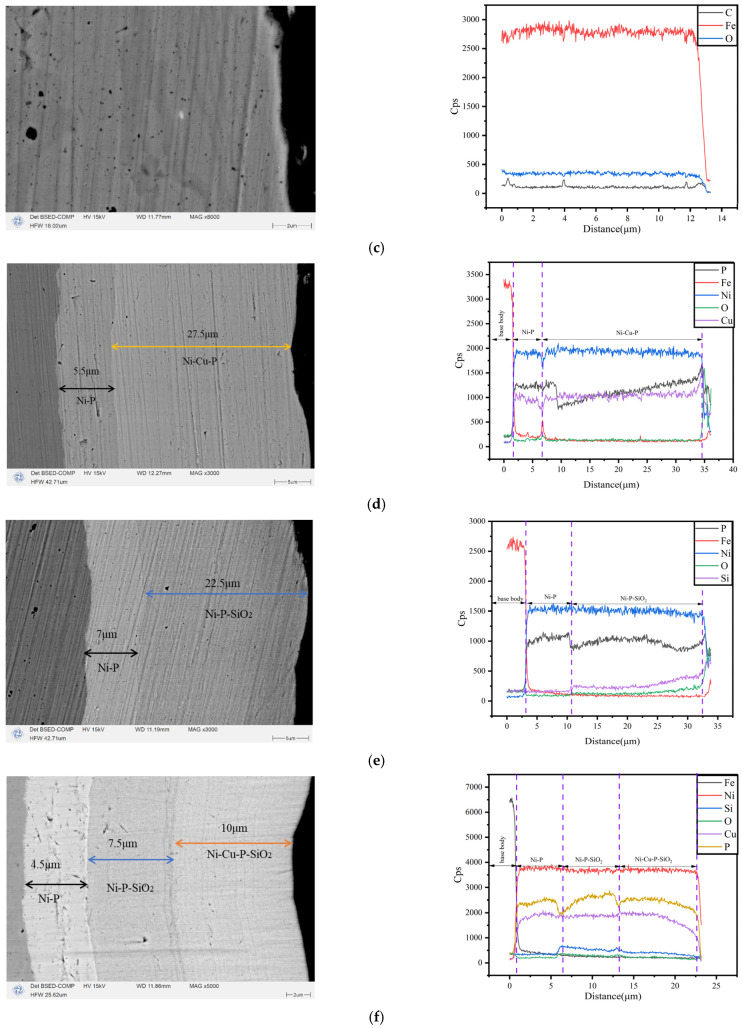

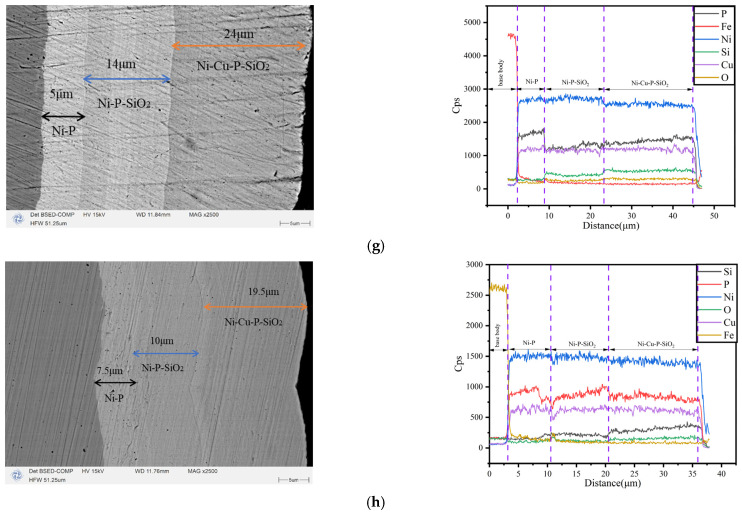
SEM and line scanning of all test samples: (**a**) ND; (**b**) Q235; (**c**) 316L; (**d**) Ni-Cu-P; (**e**) Ni-P-SiO_2_; (**f**) Ni-Cu-P-SiO_2_ (20 g/L); (**g**) Ni-Cu-P-SiO_2_ (40 g/L); (**h**) Ni-Cu-P-SiO_2_ (60 g/L).

It can be seen from Figure 15a,b that the corrosion depth of the two carbon steel surfaces is relatively uniform, but there are more multi-point corrosion pits inside the matrix, and the corrosion phenomenon is comprehensive. It can be speculated that with the increase in corrosion time, the corrosion of the surface will further deteriorate, and the exfoliation of corrosion products will promote the next round of corrosion. Compared with the two kinds of carbon steel, the corrosion damage of 316L is lighter, and the black material on the surface is produced during machining. The corresponding EDS line scan results show that the content of Fe on the surface of ND, Q235, and 316L changes sharply, indicating that the corrosion reaction on the outer surface is mainly the dissolution of Fe. The outer surface of the five coatings displays different degrees of damage. Due to the existence of pores, pitting corrosion occurs. However, due to its double-layer or three-layer composite structure, the substrate is effectively protected from corrosion. The EDS scanning results show that there is no Fe on the surface, and the content of other elements is evenly distributed, indicating that the coating still provides good protection after corrosion and does not cause damage to the substrate.

### 3.4. Analysis of the Coatings’ Anti-Corrosion Fouling Mechanisms

In summary, in the flue gas heat exchanger, the heat exchange tube bundle faces two problems: acid dew point corrosion and flue gas ash deposition. The two are interrelated, and the coating plays an important role in both processes. Based on this, we propose a coating protection model based on acid–ash coupling. According to the results presented in Section 3.3, when the CuSO_4_ concentration is 0.4 g/L and the SiO_2_ concentration is 40 g/L, the coating possesses the best comprehensive electrochemical parameters and the best corrosion resistance. A change in SiO_2_ concentration is also accompanied by a change in the surface energy of the coating: with an increase in SiO_2_ concentration, the roughness of the coating surface increases and the surface energy increases [42], which makes it easier to for the coating to adsorb other substances. According to the two-stage acid–ash coupling theory [31], in the initial stage, corrosion and ash deposition occur on the surface of the heat exchange tube bundle. At this time, due to the addition of SiO_2_, the ash adsorbed on the surface increases, and the fly ash begins to accumulate and grow on the surface due to the thermophoresis movement and gradually begins to change to the second stage. At the same time, the corrosion products begin to form on the surface. SiO_2_ exhibits excellent corrosion resistance, effectively reducing the formation of corrosion products, lowering the surface energy of the coating, and mitigating fly ash aggregation. These are opposite mechanisms of action.

In the second stage, the ash accumulates into a thick layer on the heat transfer surface. At this time, there will be a no-condensation zone, a main condensation zone, and a secondary condensation zone [8] in the ash layer due to the existence of a temperature difference. At this time, the condensation reaction of sulfuric acid vapor mainly occurs in the main condensation zone in the ash layer. The reaction on the heat transfer surface represents a low level of penetration of the condensate into the main condensation zone and the oxidation corrosion of oxygen penetrating into the surface through the gap in the ash layer. In the first stage, the increase in SiO_2_ concentration helps to establish a denser ash layer, resulting in less oxygen penetration and thus less corrosion.

Through the above two stages, it can be seen that the concentration of SiO_2_ has both promoting and inhibiting effects on ash deposition and corrosion prevention. However, according to the results in Section 3.2 for the fouling resistance and weakening heat transfer coefficient, it is found that the higher the concentration of SiO_2_, the better the heat transfer effect of the coating. When it reaches 60 g/L, the effect is the best. Therefore, we can conclude that the effect of SiO_2_ on reducing surface energy by reducing the appearance of corrosion products through better anti-corrosion characteristics is more significant than the negative effect of SiO_2_ on increasing the surface energy of the coating.

## 4. Conclusions

In this study, a monitoring system was developed to evaluate the heat exchange performance, fouling resistance, and anti-corrosive properties of different coating materials for coal-fired boilers operating under low-temperature ash deposition and dew point corrosion conditions. The objective was to investigate the fouling and corrosion issues arising from flue gas during heat exchange processes. An innovative medium-temperature alkaline chemical deposition method was employed to successfully produce a Ni-Cu-P-SiO_2_ coating, and comparisons were made with conventional Ni-Cu-P and Ni-P-SiO_2_ coatings. Following analysis of all these materials, the following conclusions were drawn:

1. The surface morphology and elemental content of the coating were analyzed by SEM and EDS. It can be observed that the surface of the coating is smooth and coherent, the plating solution is well dispersed, and there is no peeling on the whole. By adjusting the concentration of SiO_2_ in the plating solution, the SiO_2_ content in the coating can be controlled, indicating that the preparation method is repeatable.
2. The corrosion resistance of the coatings was analyzed by means of the polarization curve method, impedance spectroscopy, and hanging corrosion experiments. Among the coating materials, the corrosion voltages and current densities of the three Ni-Cu-P-SiO_2_ coatings were generally better than those of the other materials; from the AC impedance experiments, the AC impedance of the Ni-Cu-P-SiO_2_ coatings was larger, which indicated that they could slow down the corrosion more effectively in the face of acidic environments; the hanging chip corrosion experiments yielded the same conclusions. Among the coatings, the Ni-Cu-P-SiO_2_ (40 g/L) coating has the lowest average corrosion rate, which also indicates that it has the best corrosion resistance among all the coatings.
3. The performance of the above materials against ash and corrosion was evaluated by building an experimental platform to simulate the flue gas environment. The results show that the thermal resistance and heat transfer attenuation coefficient of the materials without coatings are generally worse than those with coatings; Cu improves heat transfer capacity, and SiO_2_ particles reduce corrosion and dust deposition. Therefore, the three kinds of Ni-Cu-P-SiO_2_ coating are superior to the Ni-Cu-P/Ni-P-SiO_2_ coatings in terms of their thermal resistance and heat transfer attenuation coefficient during stable operation. The results of the profile scanning proved that no significant corrosion occurred below the dew point, which is highly beneficial to the recovery of waste heat from flue gas when the coating contains 3.34 wt% Cu and 2.16 wt% SiO_2_ (corresponding to 1.01 wt% Si), confirming the synergistic effect of Cu and SiO_2_ in mitigating acid–ash coupling degradation.

## Figures and Tables

**Table 1:** Content of components (mass fraction, %).

Material	Q235	ND	316L
C	0.12–0.20	≤0.10	≤0.030
Si	≤0.30	0.20–0.40	≤1.00
Mn	0.30–0.70	0.35–0.65	≤2.00
P	≤0.045	≤0.10	≤0.045
S	≤0.045	≤0.10	≤0.030
Cu	≤0.30	0.25–0.45	-
Ni	≤0.30	-	10.00–14.00
Cr	≤0.30	0.70–1.20	16.00–18.00
Mo	-	-	2.00–3.00
Sb	-	≤0.10	-

**Table 2:** Electrolytic alkaline cleaning: composition of the bath and operating parameters.

Bath Composition	Concentration	Parameter	Value
NaOH	15–25 g/L	Temperature	20–30 °C
Na_2_CO_3_	15–25 g/L	Washing time	5–10 min
Na_3_PO_4_	20–30 g/L		
Na_2_SiO_3_	5–10 g/L		

**Table 3:** Bath components and preparation conditions of coatings.

	Ni-Cu-P	Ni-P-SiO_2_	Ni-Cu-P-SiO_2_ SiO_2_ (20 g/L)	Ni-Cu-P-SiO_2_ SiO_2_ (40 g/L)	Ni-Cu-P-SiO_2_ SiO_2_ (60 g/L)
NiSO_4_⋅6H_2_O	15–25 g/L	15–25 g/L	15–25 g/L	15–25 g/L	15–25 g/L
NaH_2_PO_2_⋅H_2_O	15–25 g/L	15–25 g/L	15–25 g/L	15–25 g/L	15–25 g/L
C_2_H_3_NaO_2_	10–15 g/L	10–15 g/L	10–15 g/L	10–15 g/L	10–15 g/L
C_6_H_5_Na_3_O_7_·2H_2_O	10–15 g/L	10–15 g/L	10–15 g/L	10–15 g/L	10–15 g/L
CuSO_4_·5H_2_O	0.4 g/L	-	0.4 g/L	0.4 g/L	0.4 g/L
Silica sol (ω = 30%)	-	40 g/L	20 g/L	40 g/L	60 g/L
pH	7.0–8.0	7.0–8.0	7.0–8.0	7.0–8.0	7.0–8.0
Temperature	65–75 °C	65–75 °C	65–75 °C	65–75 °C	65–75 °C
Intermediate Ni-P layer	Yes	Yes	Yes	Yes	Yes
Intermediate Ni-P-SiO_2_ layer	No	No	Yes	Yes	Yes

**Table 4:** Content of components.

	Ni-Cu-P		Ni-P-SiO_2_		Ni-Cu-P-SiO_2_ (20 g/L)		Ni-Cu-P-SiO_2_ (40 g/L)		Ni-Cu-P-SiO_2_ (60 g/L)	
**Element**	**Weight (%)**	**Atomic (%)**	**Weight (%)**	**Atomic (%)**	**Weight (%)**	**Atomic (%)**	**Weight (%)**	**Atomic (%)**	**Weight (%)**	**Atomic (%)**
Ni	86.66	79.53	89.01	80.78	86.18	78.55	85.42	77.65	85.42	77.34
P	10.26	17.86	9.24	15.90	10.45	18.05	10.23	17.62	9.61	16.49
Cu	3.08	2.61			2.85	2.40	3.34	2.80	3.06	2.56
Si			1.75	3.33	0.52	0.99	1.01	1.92	1.90	3.60

**Table 5:** Ultimate analysis of coals (wt%).

C	H	N	O	S
80.46	4.16	0.76	13.97	0.65

**Table 6:** Analysis on chemical composition (wt%).

K_2_O	Na_2_O	SiO_2_	Al_2_O_3_	Fe_2_O_3_	CaO	MgO	SO_3_	TiO_2_	P_2_O_5_
0.36	0.12	50.93	32.72	7.76	2.36	0.85	2.16	0.85	0.14

**Table 7:** Circuit parameter after fitting.

Sample	R_s_ (Ω·cm^2^)	R_f_ (Ω·cm^2^)	CPE_f_		R_ct_ (Ω·cm^2^)	CPE_dl_	
			Y_0_ (Ω^−1^·cm^−2^·S^n^)	n		Y_0_ (Ω^−1^·cm^−2^·S^n^)	n
Q235	2.016	/	/	/	90.97	0.000151	0.901
ND	2.370	/	/	/	9.641	0.00104	0.942
316L	2.339	/	/	/	14,357	0.000653	0.669
Ni-Cu-P	2.251	442.8	0.000744	0.823	1112	0.0155	0.656
Ni-P-SiO_2_	2.104	2106	0.000484	0.932	1710	0.001430	0.701
Ni-Cu-P-SiO_2_ (20 g/L)	2.351	351.5	0.000064	0.952	3918	0.000155	0.642
Ni-Cu-P-SiO_2_ (40 g/L)	2.213	336.5	0.000207	0.918	4130	0.000337	0.633
Ni-Cu-P-SiO_2_ (60 g/L)	2.105	1916	0.002207	0.932	1324	0.003435	0.710

**Table 8:** Error in circuit parameter fitting (%).

Sample	R_s_	R_f_	CPE_f_		R_ct_	CPE_dl_	
			Y_0_	n		Y_0_	n
Q235	0.875	/	/	/	1.382	0.935	0.556
ND	0.396	/	/	/	0.585	2.453	0.431
316L	2.416	/	/	/	8.812	1.333	0.605
Ni-Cu-P	0.656	5.986	0.777	0.591	3.661	3.267	2.213
Ni-P-SiO_2_	0.642	8.419	0.681	0.396	8.855	8.226	15.178
Ni-Cu-P-SiO_2_ (20 g/L)	0.879	4.606	0.994	0.802	3.222	3.179	1.663
Ni-Cu-P-SiO_2_ (40 g/L)	0.508	2.732	0.577	0.432	1.028	1.843	1.874
Ni-Cu-P-SiO_2_ (60 g/L)	0.533	10.851	0.671	0.404	9.552	9.032	4.904

**Table 9:** Electrochemical results for each surface material.

Sample	E_corr_ (V, Mean)	Standard Deviation	I_corr_ (A·cm^−2^, Mean)	Standard Deviation
ND	−0.343	5.888 × 10^−3^	5.776 × 10^−3^	4.360 × 10^−4^
Q235	−0.424	5.907 × 10^−3^	3.562 × 10^−3^	4.825 × 10^−4^
316L	0.527	1.70 × 10^−3^	1.707 × 10^−5^	2.704 × 10^−6^
Ni-Cu-P	0.106	9.031 × 10^−3^	4.220 × 10^−5^	8.300 × 10^−6^
Ni-P-SiO_2_	0.036	5.888 × 10^−3^	1.038 × 10^−5^	6.341 × 10^−7^
Ni-Cu-P-SiO_2_ (20 g/L)	0.049	1.167 × 10^−2^	1.894 × 10^−5^	5.628 × 10^−6^
Ni-Cu-P-SiO_2_ (40 g/L)	0.033	2.494 × 10^−3^	2.287 × 10^−5^	8.523 × 10^−6^
Ni-Cu-P-SiO_2_ (60 g/L)	−0.023	1.476 × 10^−2^	1.417 × 10^−5^	2.278 × 10^−6^

**Table 10:** Corrosion rate of all samples.

Sample	Corrosion Rate (mm/a)	Standard Deviation
ND	12.82	0.69
Q235	15.83	0.71
316L	0.03	0.01
Ni-Cu-P	0.34	0.04
Ni-P-SiO_2_	0.1	0.01
Ni-Cu-P-SiO_2_ (20 g/L)	0.09	0.01
Ni-Cu-P-SiO_2_ (40 g/L)	0.03	0.02
Ni-Cu-P-SiO_2_ (60 g/L)	0.08	0.02

## Data Availability

The raw data supporting the conclusions of this article will be made available by the authors on request.
